# “Stay in touch while on the bench” - how the MIQE applet can increase the quality of your qPCR and dPCR experiments

**DOI:** 10.1186/1471-2164-15-S2-P15

**Published:** 2014-04-02

**Authors:** Afif M Abdel Nour, Esam Azhar, Michael W Pfaffl, Ghazi Damanhouri

**Affiliations:** 1 Special Infectious Agents Unit, King Fahd Medical Research Center, King Abdulaziz University, Jeddah, Kingdom of Saudi Arabia; 2 Medical Laboratory Technology Department, Faculty of Applied Medical Sciences, King Abdulaziz University, Jeddah, Kingdom of Saudi Arabia; 3 Physiology Weihenstephan, Center of Life and Food Sciences, Weihenstephan, Germany; 4 King Fahd Medical Research Center, King Abdulaziz University, Jeddah, Kingdom of Saudi Arabia

## Background

How to check the quality of your qPCR experiments or the reliability of an international publication by an electronic device? From now on iOS based mobile devices, e.g. iPhone, iPad or iPod, will help you to increase the quality of qPCR experiment or publication, by providing a ‘MIQE (The MIQE Guidelines - **M**inimum **I**nformation for publication of **Q**uantitative real-time PCR **E**xperiments) qPCR’ applet [1,2]. Three years ago we created the first interactive solution for scientific guidelines, based on the MIQE qPCR publication [3].

## Materials and methods

This first applet was downloaded 7800 times from 89 different countries, and recently Biotechniques journal has selected the APP as one of the leading “methods-oriented applets” and recommended it by the “websites every life scientist should try”.

## Results

**Figure 1:**
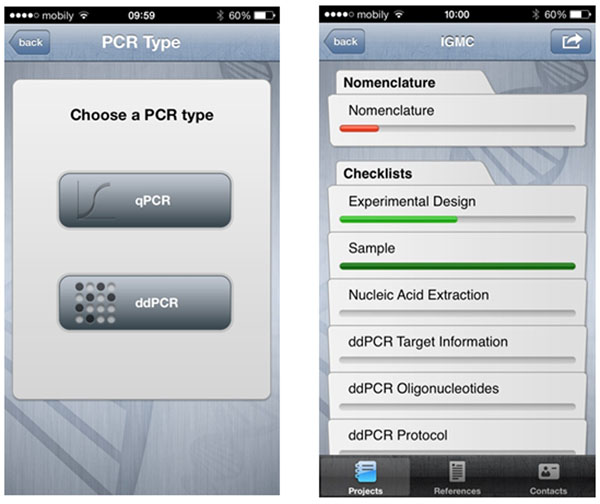
Screenshots from the iOS applet.

After this great success we are presenting today the MIQE applet for the digital PCR (Figure 1).

The digital PCR MIQE guidelines were published recently by a group of experts [4]. This new application could be used by scientists to check whether their digital PCR (dPCR) experiment or the used literature fulfills the MIQE requirement. The ‘MIQE Guidelines’ checklist provides 83 parameters that dPCR studies should be required or recommended to meet before being considered for publication. This checklist is based on the original published MIQE checklist for the dPCR and we hope it will increase future publication quality and reliability.

## Conclusions

There are much more wider seen goals of the ‘MIQE guidelines’, all in all the goals might be summarized as follow: 1) to increase reliability of results, 2) to help insuring the integrity of scientific work, with major focus on biological relevance. This is an easy to use applet that can help reviewers and authors to gain time in there manuscript preparation. Recently by using this applet we managed to analyze and evaluate 461 scientific papers published by Arabian countries (paper accepted in PLOSOne).
